# Comparison of Two Different Analgesic Prescription Strategies and Healthcare Systems: Slovenia vs. the Netherlands

**DOI:** 10.3389/fpain.2021.723797

**Published:** 2021-08-27

**Authors:** Ajda Bedene, Anita Strmljan, Eveline L. A. van Dorp, Mitja Udovič, Willem M. Lijfering, Marieke Niesters, Frits R. Rosendaal, Albert Dahan, Jurij Fürst

**Affiliations:** ^1^Department of Clinical Epidemiology, Leiden University Medical Center, Leiden, Netherlands; ^2^Department of Anesthesiology, Leiden University Medical Center, Leiden, Netherlands; ^3^Health Insurance Institute of Slovenia, Ljubljana, Slovenia

**Keywords:** pain medication, opioid epidemic, pain management, prescription guidelines, European guidelines

## Abstract

**Background:** Prescribing practice of pain medication is changing in the Netherlands; opioids are used more often instead of nonsteroidal anti-inflammatory drugs (NSAIDs), therefore we aimed to compare the use of pain medication with Slovenia which has stringent prescribing rules for strong opioids.

**Methods:** We conducted a cohort study into national prescription databases of the Netherlands and Slovenia covering pharmacy claims between January 1, 2013 and December 31, 2019. In the analysis about 17 million Dutch and 2 million Slovenian residents were included.

**Findings:** The use of opioids and NSAIDs was higher in Slovenia than in the Netherlands. More frequent use of opioids in Slovenia could be almost entirely explained by weak opioids (about 6% of the population), whereas they were prescribed 50% less frequently in the Netherlands. The opioid use has increased by about 20% in the Netherlands (4.85 and 6.00% of the population in 2013 and 2018, respectively), and the majority of this increase could be explained by strong opioids (4.05% in 2018), specifically, by oxycodone whose use increased by more than 2-fold between 2013 and 2019. In comparison, oxycodone was seldomly used in Slovenia (about 0.3% of the population received a prescription in a year).

**Interpretation:** When medication use is controlled by stringent prescribing rules, like for strong opioids in Slovenia, the use is lower as compared to when such rules do not exist.

## Introduction and Rationale

The use of opioids has become wide-spread worldwide and the number of opioid overdoses have risen to such numbers that some countries proclaimed an opioid epidemic (1). Causes of this increase in opioid use are not well known, but are probably multifactorial. Remarkably, the situation regarding opioid crisis differs between countries, and a probable reason for this is lack of harmonized pain relief guidelines. In 1996, the World Health Organization (WHO) published a revised guideline about the treatment of pain relief in patients with cancer, wherein the now established three-step pain ladder was introduced, which entails a stepwise approach to pain relief, starting with acetaminophen/paracetamol and ending, *via* nonsteroidal anti-inflammatory drugs (NSAIDs) and opioids for mild to moderate pain, at opioids for moderate to severe pain as a last resort (2, 3). As a response to the uncontrolled rate of opioid overdoses in the United States, a new guideline by the Centers for Disease Control and Prevention was proposed in 2016, that provides recommendations regarding safety of opioid use in the treatment of chronic non-cancer pain (4). A similar approach was taken by the National Institute For Health And Care Excellence that announced a new guideline for chronic pain in 2021, but has not yet been published (5).

In the Netherlands, physicians follow the WHO pain ladder. The guide is supplemented by the pain standard of the general practitioners' society in the Netherlands, and the postoperative pain guideline that was revised in 2013. Since then, the prevalence of opioids and NSAIDs use has changed in the Netherlands. It has been previously reported by our group and others, that the opioid prescription prevalence increased from 814,211 individuals in 2013 to 1,027,019 individuals in 2017 who registered to at least one opioid prescription per calendar year, (6, 7) while the number of individuals with NSAIDs prescriptions has decreased by *n* = 255,675 individuals between 2013 and 2017 (8). Based on the scientific literature it has been evident for some time that the use of NSAIDs is associated with increased risk of gastrointestinal, cardiac and renal complications (9–14), which may have encouraged physicians against NSAIDs prescribing. Moreover, in the 2013 edition of the Dutch postoperative pain guideline, special attention was given to novel opioid analgesic medications with oxycodone being one of them. The working group recommended prescribing morphine and piritramide in treatment of moderate to severe postoperative pain, but also oxycodone when oral intake is possible (9). This advice may have encouraged Dutch physicians to consider oxycodone as a pain treatment option.

In Slovenia, physicians also follow national guidelines on non-cancer and cancer pain (15, 16), which were based on the WHO pain ladder. The prevalence of analgesic prescriptions is routinely checked by the National Institute of Public Health for surveillance purposes (17). In addition to this guideline, there are special prescribing rules that concern only “strong” opioids, which we define as all registered opioid medications that do not contain tramadol. In other words, “weak” opioids are those opioids that contain either tramadol or tramadol in combination with paracetamol, for which special prescribing rules do not apply. These special prescribing rules are: special hand-written prescription form in a duplicate, compulsory identification both at the doctor's office and in the pharmacy and required age more than 18 years to be able to fill the medication, prescription of the amount that lasts up to 30 days of persistent use, repeat prescription prohibited.

In the current study, we hypothesized that the prevalence of opioid use is lower in Slovenia than in the Netherlands, because of this strict prescription policy regarding strong opioids (18). In contrast, we expected that the use of NSAIDs is higher in Slovenia compared with the Netherlands, because prescribing restrictions that pertain to strong opioids in Slovenia do not apply to this group of analgesic medication. Therefore, we set out to compare the prevalence of analgesic medications use in the total population of Slovenia and in the Netherlands between 2013 and 2019.

## Methods

### Setting and Participants

We conducted a nation-wide cohort study for which we analyzed national prescription datasets from the Netherlands and from Slovenia. Vital statistics of the Netherlands are managed by Statistics Netherlands, that collects information on all residents (about 17 million people). Prescription data of Slovenia are collected and managed by the Health Insurance Institute of Slovenia. In this dataset the whole population of Slovenia is covered which is about 2 million people. In this cohort study, we investigated data that pertain to the time between January 1st, 2013 and December 31st, 2019.

This study was exempt from the Medical Ethical Review Committee of Leiden University Medical Center (reference number: G21.033), as well as from the National Medical Ethics Committee of Slovenia after a review (reference number: 0120-17/2021-3). All personal information of participants in the Netherlands was identified by third parties prior to analysis. Authorized employee (M.U.) of the Health Insurance Institute of Slovenia had access to personal information of participants, and prepared identified aggregated data prior to analysis. This ensures that no personal information can be disclosed from the results.

### Data Sources

#### The Netherlands

##### Statistics Netherlands

Prescription reimbursement data were collected for all Dutch residents entitled to pharmaceutical care, i.e., those insured by the basic health insurance which is mandatory by law and covers almost all residents, *n* = 17,163,404 (99.9%) in 2018 (19). The Health Care Institute of the Netherlands collects prescription reimbursement data and provides it to Statistics Netherlands. Medication dispensed from outpatient, community pharmacies, and in residential homes for elderly are collected in the national reimbursement database, whereas medicine use in hospitals and in nursing homes is not collected (20). In the prescription reimbursement database of Statistics Netherlands medications are classified according to the Anatomical Therapeutic Chemical Classification System (ATC) (21), and are made available on the 3rd level (4 position) of the ATC code. These data were at the time of the analysis (in December 2020) published up to and including 2019.

##### Medicine and Medical Devices Information Project (GIP)

Prescription reimbursement data does not contain information on the level of active substances, i.e., 5th level of the ATC classification, therefore we analyzed the open source prescription data (GIP) provided by the Health Care Institute of the Netherlands (22). The Institute is responsible for the content of the GIP data, keeping the data updated as well as its accuracy (23). The GIP data contains information on all medications reimbursed under the basic health insurance (24). The information that is publicly available on the GIP database may be used as desired, when the source of the information is declared (25).

#### Slovenia

Prescription data were collected for all residents of Slovenia entitled to the pharmaceutical care which is insured by the national health insurance that covered almost all residents (about 2 million, 99.97%) throughout the observation time. Prescription data records all medications dispensed from community pharmacies. Medicines used during hospitalization and during outpatient hospital or nursing home encounter are not recorded in this dataset. Note that magistral preparations containing opioids are not recorded in this dataset. All prescriptions for medications were identified based on the 5th level of the ATC classification.

### Variables and Outcomes

We performed an analysis into national vital statistics of the Netherlands and of Slovenia, in which all citizens who resided in an individual country at the time of observation, i.e., between January 1st, 2013 and December 31st, 2019, were included. To obtain information on national vital statistics data we utilized publicly available data in both countries. Information on age (stratified into age groups) and sex for the Netherlands was obtained from “StatLine” of Statistics Netherlands (26), and the same information for Slovenia from Statistical Office of the Republic of Slovenia (27).

We identified individuals who received a prescription for a medication and also filled the prescription in a pharmacy. The number of those who received at least one prescription for an analgesic medication in a calendar year was used to calculate annual prevalence, which is the main outcome of this study. We investigated two analgesic medication groups that are represented in the WHO pain ladder, namely opioids, and NSAIDs. Opioid prescriptions were identified based on the ATC code N02A, and NSAIDs prescriptions based on the ATC code M01A. There are substantial differences in the availability of individual active substances in Slovenia and in the Netherlands, however, we classified opioid medications as “strong” and “weak”, based on tramadol. When a medication contained tramadol, it was classified as a weak opioid, and otherwise as a strong opioid medication. These opioid groups were defined based on “Medicinal Products Act” in Slovenia, in order to compare the two countries. A comprehensive list of all registered active substances is available in the Supplementary Table 1.

### Statistical Methods

We performed a descriptive analysis of the total population in the Netherlands, in Slovenia and in the European Union between 2013 and 2019, and calculated the total number of residents living in each individual country. Then, we stratified the total population of each individual country by age, which was grouped into five age categories: from 0 to 14 years, from 15 to 24 years, from 25 to 44 years, from 45 to 64 years and more than 65 years, and sex. These results were presented as total numbers and as a proportion of the total population. Then, we identified the number of individuals to whom opioids, and NSAIDs were prescribed and calculated an annual prevalence percentage with corresponding 95% confidence interval (CI) for each individual country through the observation period. To explore time-trends of opioids, and NSAIDs prescriptions in each individual country we calculated relative risks (RR) with corresponding 95% CI in which we selected the calendar year 2013 as a reference. In order to make the annual prevalence calculations as well as the time-trend analysis comparable between the Netherlands and Slovenia, we corrected for demographic differences (age and sex) between these two countries with direct standardization where we utilized the population of European Union of 2013 as weights. We presented results of the latter analysis as standardized prevalence percentage with corresponding 95% CI, and standardized RR with corresponding 95% CI where we took the calendar year of 2013 as a reference. There were no individuals lost to follow-up nor were any data lost in the merging process.

All statistical analyses were performed with SPSS for Windows, release 25.0 (SPSS, Chicago, IL, USA). Figures were created with R studio (A Language and Environment for Statistical Computing, R Core Team, R Foundation for Statistical Computing, Vienna, Austria, https://www.R-project.org), using R package ggplot2 version 3.2.125 (28). The STROBE statement checklist for cohort studies was used to guide reporting of the findings.

## Results

### Participants

In the analysis, all residents of the Netherlands and Slovenia were included. There were *n* = 2,080,908 individuals registered in Slovenia in 2019. Of these, about a half were women (*n* = 1,042,252) (Table 1). The age structure was similar in both countries as 47.2% of the Dutch population of 2019 (*n* = 17,282,163), and 48.5% of the population of Slovenia of 2019 was older than 45 years. Women accounted for about 50% of the total Dutch population and of the Slovenian population throughout the observation period (Table 1). Demographic characteristics of both Slovenia and the Netherlands are similar to the population of European Union of 2013 that was selected to standardize the annual prevalence of different analgesic medications.

**Table 1:** Population characteristics, the Netherlands, Slovenia, and the total population of European Union, from 2013 to 2019.

	**2013, No. (%)**	**2014, No. (%)**	**2015, No. (%)**	**2016, No. (%)**	**2017, No. (%)**	**2018, No. (%)**	**2019, No. (%)**
**Slovenia**							
Total	2,058,821	2,061,085	2,062,874	2,064,188	2,065,895	2,066,880	2,080,908
Age groups, years							
0–14	298,095 (14.48)	301,053 (14.61)	304,310 (14.75)	306,390 (14.84)	308,594 (14.94)	310,677 (15.03)	313,706 (15.08)
15–24	215,937 (10.49)	208,493 (10.12)	202,709 (9.83)	199,154 (9.65)	195,820 (9.48)	194,130 (9.39)	194,795 (9.36)
25–44	595,959 (28.95)	592,346 (28.74)	586,705 (28.44)	581,084 (28.15)	574,065 (27.79)	565,162 (27.34)	563,159 (27.06)
45–64	596,685 (28.98)	599,087 (29.07)	599,764 (29.07)	597,458 (28.94)	596,990 (28.90)	595,649 (28.82)	596,194 (28.65)
>65	352,145 (17.10)	360,106 (17.47)	369,386 (17.91)	380,102 (18.41)	390,426 (18.90)	401,262 (19.41)	413,054 (19.85)
Sex							
Women	1,039,760 (50.50)	1,040,211 (50.47)	1,040,645 (50.45)	1,040,855 (50.42)	1,040,770 (50.38)	1,039,839 (50.31)	1,042,252 (50.09)
Men	1,019,061 (49.50)	1,020,874 (49.53)	1,022,229 (49.55)	1,023,333 (49.58)	1,025,125 (49.62)	1,027,041 (49.69)	1,038,656 (49.91)
**The Netherlands**							
Total	16,779,575	16,829,289	16,900,726	16,979,120	17,081,507	17,181,084	17,282,163
Age groups, years							
0–14	2,877,922 (17.15)	2,850,074 (16.94)	2,827,066 (16.73)	2,799,772 (16.49)	2,781,768 (16.29)	2,762,624 (16.08)	2,739,819 (15.85)
15–24	2,049,538 (12.21)	2,058,275 (12.23)	2,070,025 (12.25)	2,084,673 (12.28)	2,101,648 (12.30)	2,116,813 (12.32)	2,131,944 (12.34)
25–44	4,333,861 (25.83)	4,287,658 (25.48)	4,242,279 (25.10)	4,217,738 (24.84)	4,214,276 (24.67)	4,222,614 (24.58)	4,255,450 (24.62)
45–64	4,693,909 (27.97)	4,714,258 (28.01)	4,753,671 (28.13)	4,791,629 (28.22)	4,824,155 (28.24)	4,839,917 (28.17)	4,840,946 (28.01)
>65	2,824,345 (16.83)	2,919,024 (17.34)	3,007,685 (17.80)	3,085,308 (18.17)	3,159,660 (18.50)	3,239,116 (18.85)	3,314,004 (19.18)
Sex							
Women	8,472,236 (50.49)	8,494,904 (50.48)	8,527,868 (50.46)	8,561,985 (50.43)	8,606,405 (50.38)	8,654,043 (50.37)	8,701,077 (50.35)
Men	8,307,339 (49.51)	8,334,385 (49.52)	8,372,858 (49.54)	8,417,135 (49.57)	8,475,102 (49.62)	8,527,041 (49.63)	8,581,086 (49.65)
**European union**							
Total	505,163,008	507,235,091	508,520,205	510,181,874	511,378,572	512,372,000	513,471,676
Age groups, years							
0–14	79,062,512 (15.65)	79,237,403 (15.62)	79,315,276 (15.60)	79,444,690 (15.57)	79,650,613 (15.58)	79,768,888 (15.57)	79,747,760 (15.53)
15–24	58,155,625 (11.51)	57,492,742 (11.33)	56,860,113 (11.18)	56,488,882 (11.07)	55,937,539 (10.94)	55,439,958 (10.82)	55,182,710 (10.75)
25–44	138,653,493 (27.45)	137,929,625 (27.19)	136,997,167 (26.94)	136,448,198 (26.75)	135,513,904 (26.50)	134,679,168 (26.29)	133,997,808 (26.10)
45–64	137,344,767 (27.19)	138,548,592 (27.31)	139,343,781 (27.40)	140,066,955 (27.45)	140,798,128 (27.53)	141,343,811 (27.59)	141,781,802 (27.61)
>65	91,946,611 (18.20)	94,026,729 (18.54)	96,003,868 (18.88)	97,733,149 (19.16)	99,478,388 (19.45)	101,140,175 (19.74)	102,761,596 (20.01)
Sex							
Women	258,781,338 (51.23)	259,724,441 (51.20)	260,301,222 (51.19)	260,886,459 (51.14)	261,414,441 (51.12)	261,841,054 (51.10)	262,332,762 (51.09)
Men	246,381,670 (48.77)	247,510,650 (48.80)	248,218,983 (48.81)	249,295,415 (48.86)	249,964,131 (48.88)	250,530,946 (48.90)	251,138,914 (48.91)

### Annual Prevalence of Opioids and NSAIDs Prescription

**Figure 1:**
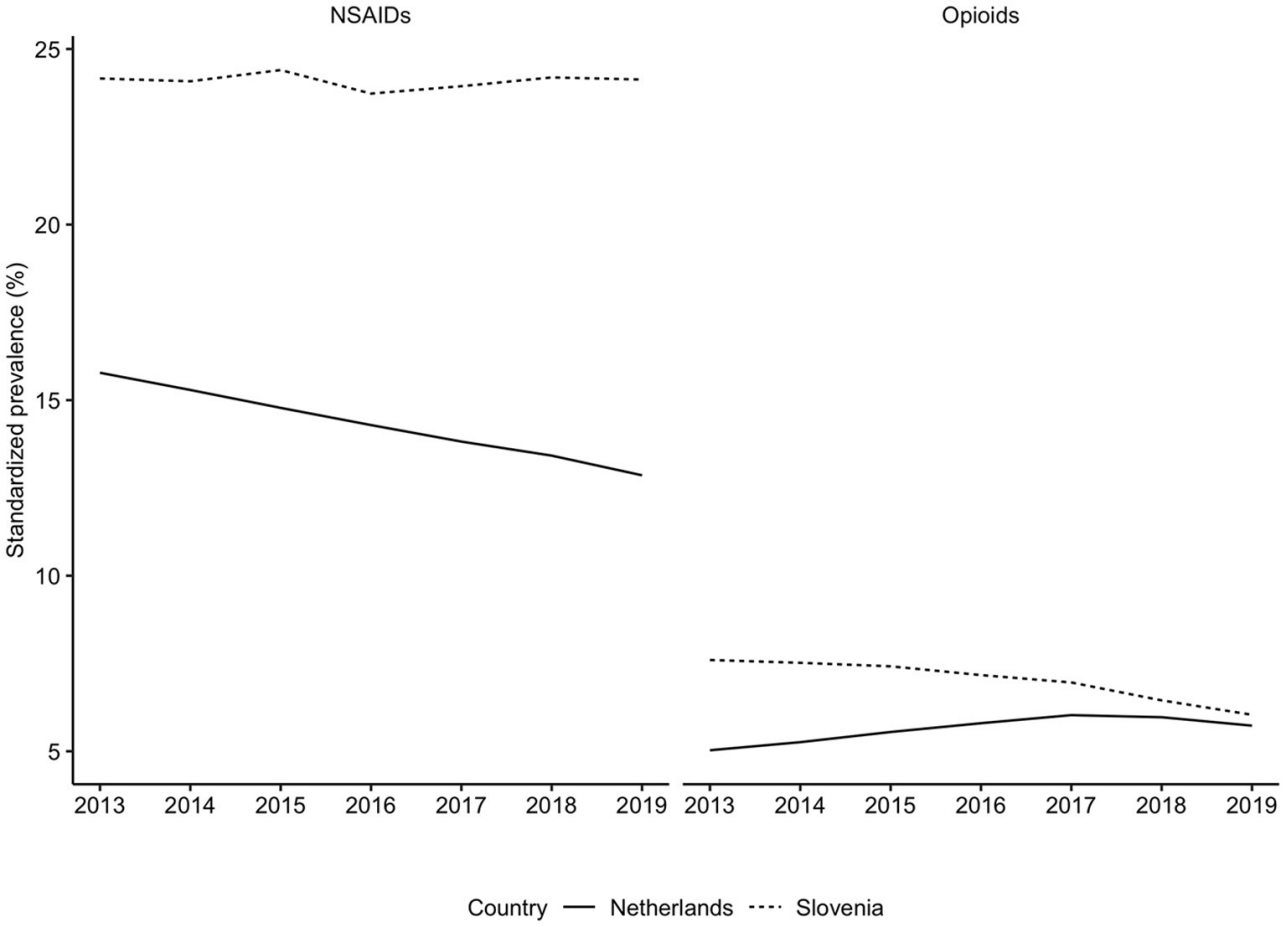
Standardized prevalence of opioids and NSAIDs use in the Netherlands and in Slovenia, from 2013 to 2019. NSAIDs, nonsteroidal anti-inflammatory drugs. Opioids were identified based on the ATC code N02A, NSAIDs based on the M01A. Prevalence was corrected for age- and sex- differences between Slovenia in the Netherlands with direct standardization where we utilized the population of the European Union of 2013 as weights.

**Figure 2:**
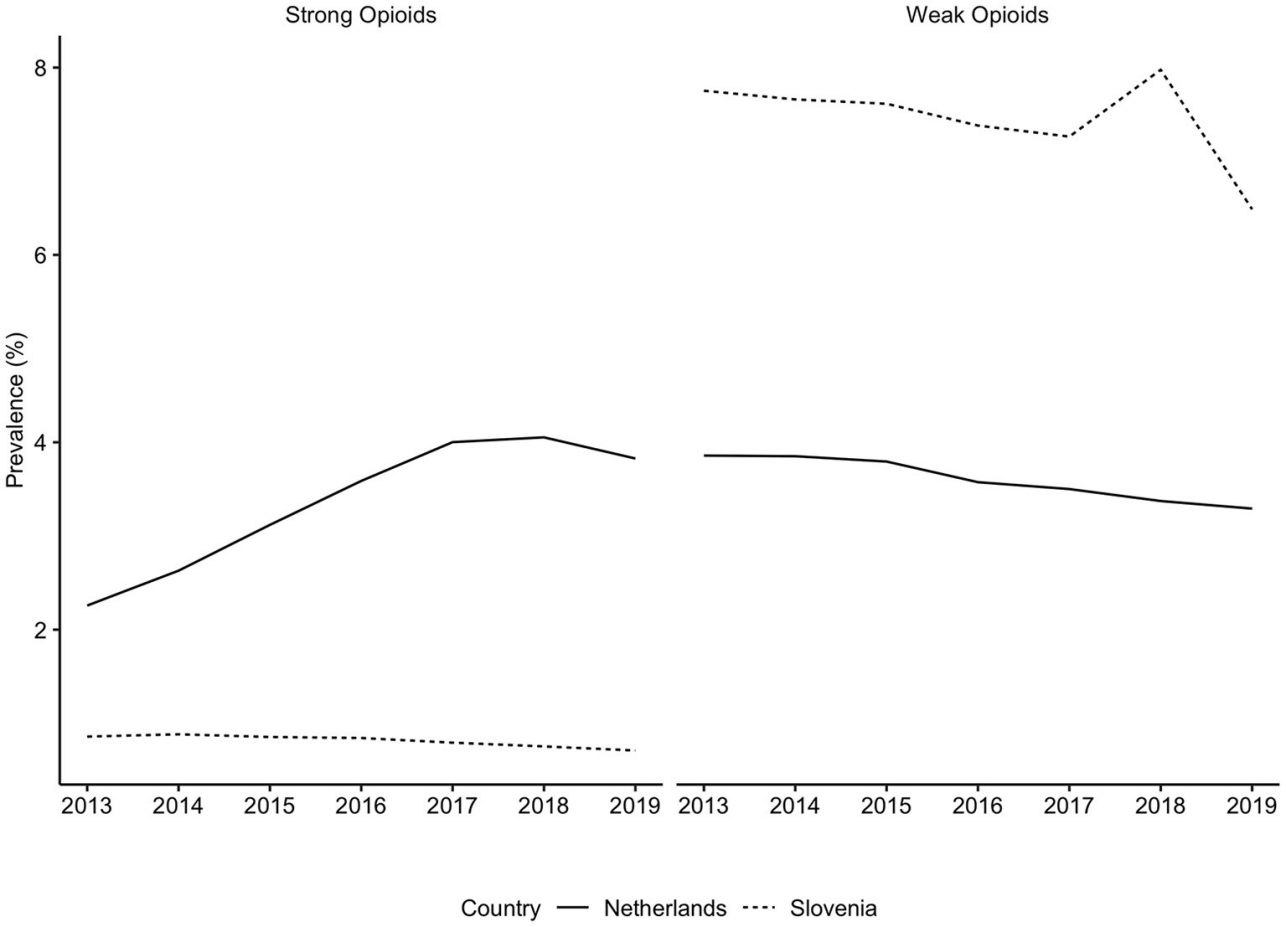
Prevalence of strong and weak opioids use in the Netherlands and in Slovenia, from 2013 to 2019. Opioids were identified based on the ATC code N02A, strong opioids were defined as all opioids except tramadol. There are differences in the availability of individual substances in each country. These differences can be found in the Supplementary Table 1.

**Figure 3:**
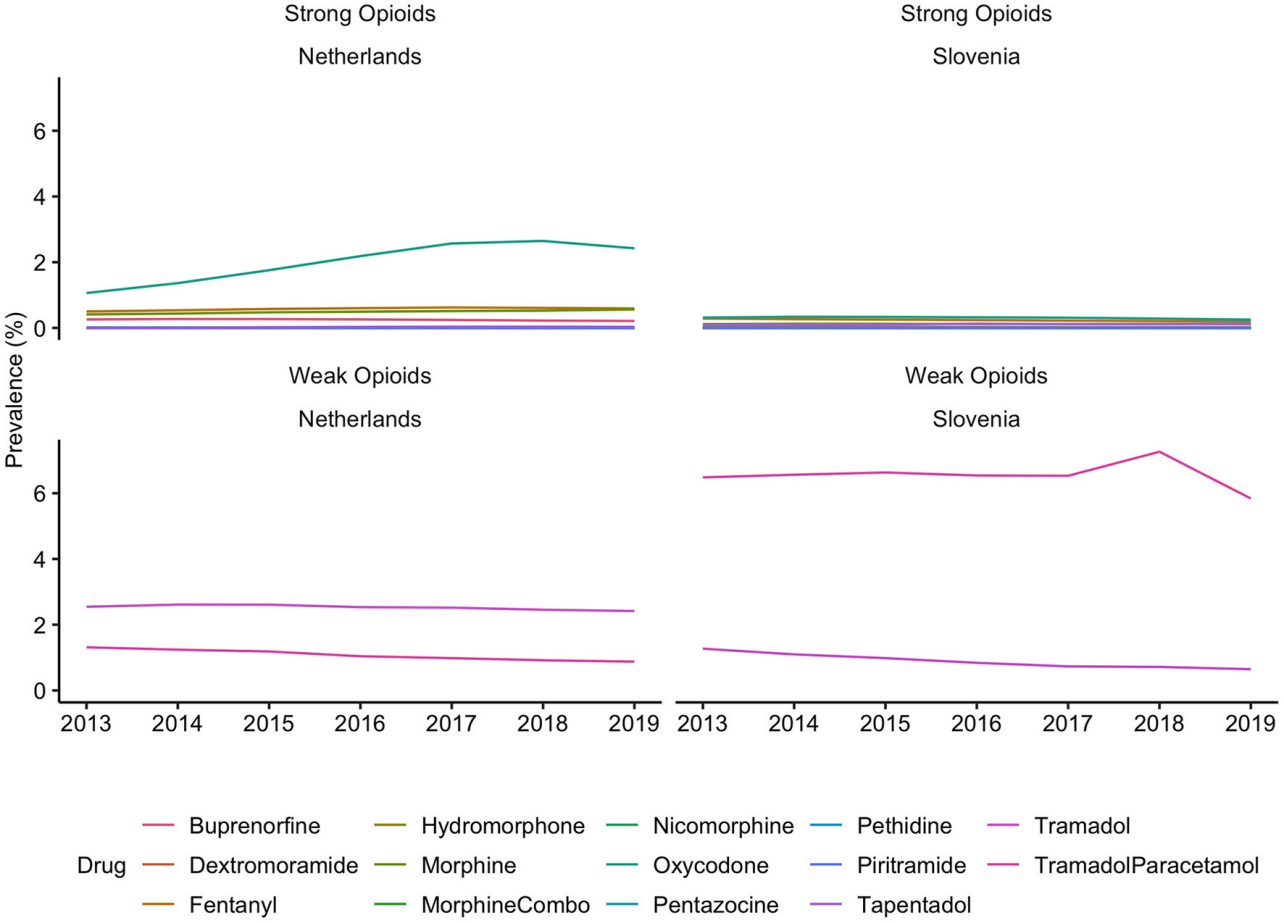
Prevalence of individual opioids use in the Netherlands and in Slovenia, from 2013 to 2019. Opioids were identified based on the ATC code N02A, strong opioids were defined as all opioids except tramadol. There are differences in the availability of individual substances in each country. These differences can be found in the Supplementary Table 1.

Generally, Slovenian residents received more pain medication compared to residents of the Netherlands (Figure 1). In Slovenia, 6.79% [95% CI, 6.75–6.82] of residents received at least one prescription for an opioid in 2018, which was 6.00% [95% CI, 5.99–6.01] in the Netherlands in the same calendar year. However, prescription opioid use is decreasing in Slovenia (standardized RR, 0.85 [95% CI, 0.84–0.85], comparing 2018 with 2013). In the Netherlands prescription opioid use is increasing over the time frame (standardized RR, 1.19 [95% CI, 1.18–1.19], comparing 2018 with 2013) (Figure 1, Supplementary Table 2). The more frequent use of prescription opioids in Slovenia could be almost entirely explained by weak opioids (~6%), whereas in the Netherlands weak opioids were less frequently prescribed (~3%) (Figures 2, 3). The majority of the increase in prescription opioid use in the Netherlands could be explained by strong opioids (RR, 1.70 [95% CI, 1.69–1.70]), specifically, by oxycodone that was prescribed to about 2% Dutch residents in 2019 (Figure 3). The prevalence of oxycodone prescription increased more than 2-fold between 2013 and 2019 in the Netherlands. In comparison, oxycodone was barely used in Slovenia throughout the observation period (about 0.3% of the population received a prescription for oxycodone in a year's time).

There were also differences between these two countries when comparing NSAIDs use (Figure 1). In 2018, about 25% of the Slovenian population and about 13% of the Dutch population received at least one prescription for NSAIDs medication (Figure 1, Supplementary Table 2). In the Netherlands the use of NSAIDs prescriptions has decreased since 2013 (standardized RR, 0.85 [95% CI, 0.85–0.85], comparing 2018 with 2013), whereas in Slovenia it remained unchanged throughout the observation time (standardized RR, 1.00 [95% CI, 1.00–1.01], comparing 2018 with 2013) (Figure 1, Supplementary Table 2).

## Discussion

In this analysis we set out to compare the annual prevalence of pain medication use in Slovenia and in the Netherlands between January 1st, 2013 and December 31st, 2019. In order to make the comparison between these two countries accurate, we corrected pain medication use for demographic differences (age and sex) with direct standardization. We discovered that the annual prevalence of opioids, and NSAIDs, was higher in Slovenia compared with the Netherlands throughout the observation period. However, strong opioid use trends investigated between 2013 and 2019 pointed in the opposite direction when these two countries were compared.

Throughout the observation period, opioid use in Slovenia has decreased between 2013 and 2019 (standardized RR, 0.80 [95% CI, 0.79–0.80], 2019 compared with 2013, prevalence of opioid use in the general population was 6% in 2019), which could be in its entirety explained by a decrease in prescription of tramadol in combination with acetaminophen/paracetamol (*n* = 121,534, 5.84%). In the Netherlands the use of opioids has increased by 20% between 2013 and 2017 and plateaued out in 2018 (standardized RR, 1.19 [95% CI, 1.18–1.19] when comparing 2018 with 2013), and the prevalence of opioid use in the general population was 6% in 2018. The increase in opioid prescription in the Netherlands can be explained almost entirely by oxycodone (*n* = 418,707, 2.42% in 2019) and tramadol (*n* = 417,649, 2.42% in 2019) use. However, the use of tramadol has been steadily decreasing since 2013 (RR, 0.95 [95% CI, 0.95–0.95] comparing 2019 with 2013), whereas the use of oxycodone increased more than 2-fold (RR, 2.28 [95% CI, 2.27–2.29], comparing 2019 with 2013). Approximately the same proportion of residents received an opioid prescription in Slovenia in 2019 as in the Netherlands in 2018. This finding is in contrast with our hypothesis, where we expected that the use of opioid medications would be higher in the Netherlands than in Slovenia.

The analysis into individual opioid medications revealed that prescription of weak and strong opioids differed between countries. The following reasons can potentially explain these findings: First, in Slovenia prescribing of strong opioids is strictly regulated by the Medicinal Products Act and requires a special prescription form. This procedure is rather complicated and time-consuming, i.e., it needs to be in a paper format, either hand-written or printed, and an entry in the book of narcotics needs to be made, which ensures full traceability of the prescribed opioid (10, 18). In Figures 2, 3 we demonstrated that almost all opioid prescriptions in Slovenia can be explained by prescribing tramadol in combination with acetaminophen/paracetamol, which is a weak opioid (also in a lower dose) and therefore not strictly regulated. In contrast in the Netherlands, opioid prescription is not as strictly regulated compared to Slovenia with less time-consuming regulations. This suggests that applying strict prescription rules for strong opioids may lead to a lower prescription rate of strong opioids.

Second, in the Netherlands the prescription of strong opioids, especially oxycodone, is recommended as demonstrated on the example of the revised postoperative pain guideline (9). This suggests that the threshold for receiving a prescription for a strong opioid is lower in the Netherlands compared to Slovenia. Additionally, many patients who receive tramadol experience gastrointestinal disturbances (29), which may have inspired Dutch physicians to prescribe less tramadol while at the same time oxycodone was advertised as a safer opioid option (30); the use of oxycodone skyrocketed and the use of tramadol plateaued (31).

Third, the difference in opioid prescription can be explained by the difference between countries in the quantity and duration of the prescribed opioids. In Slovenia, physicians are not allowed to prescribe strong opioids for longer than 30 days. In contrast, there are no restrictions on the length of dosing imposed in the Netherlands (10, 18). Prescribing a strong opioid on repeat prescription enables a patient to have a continuous prolonged access to the opioid medication without consulting with a medical professional. Although, the pain guideline of the general practitioners' society in the Netherlands advises on evaluation of opioid use every 1–2 weeks (10), 16.8% of patients still received a prescription for a strong opioid for more than 90 days of consistent use (32).

We also observed differences in the use of NSAIDs between the two countries. Every one in four residents in Slovenia and about one in seven residents in the Netherlands received at least one prescription for NSAIDs medication in a year's time. The number of individuals to whom NSAIDs were prescribed has steadily decreased for the past decade in the Netherlands, while their use in Slovenia remained stable. A possible explanation for this could be that in the Netherlands physicians put greater emphasis on their unfavorable adverse events profile (8, 33), as well as advise patients' to buy NSAIDs over-the-counter since the most clinically useful strength, 400 mg, is not reimbursed by the basic health insurance (24). Furthermore, the increase in prescriptions of strong opioids may have led to less indications to prescribe NSAIDs.

To fully understand differences between Slovenia and the Netherlands we must also explore differences in healthcare systems. In Slovenia there is a great emphasis on prevention and complementary medicine, for example physical therapy, including exercise, hydro therapy, and psychological support (34). In general, it is more acceptable to make use of treatments that may not be as cost effective in pain relief and may take longer time as compared to taking a pill, but they are in fact more patient-friendly. This is as opposed to the Netherlands where the healthcare system is cost-driven and this holistic approach has been partly cut from the healthcare budget (35). Additionally, in the Netherlands standards of hospital care among others include level of pain as perceived by hospitalized patients. This means, that hospitals, according to a survey were able to keep their patients' pain levels low, were awarded with better rating compared to those hospitals where patients experienced more pain while hospitalized (36). Hence, to achieve better hospital performance Dutch physicians may prescribe more strong pain medication to efficiently combat pain.

This research has some methodological issues that warrant a comment. First, we have no information about the indication for which the medication was prescribed, the amount, dose, nor for how long the medication was used. Therefore, calculation of defined daily doses as well as morphine milligram equivalents is not possible. Second, there may be other discrepancies, measured and unmeasured, between countries that could further explain differences in the use of pain relief medication, however such information is not known to us. Third, we do not have information on over-the-counter medication use, therefore use especially of NSAIDs is most probably underestimated. Opioids are in general not available as an over-the-counter medication; the only exception is codeine that can be bought as pain medication in small doses in Slovenia, and is available as antitussive medication in the Netherlands.

In conclusion, the use of strong opioids is increasing in the Netherlands and it is decreasing in Slovenia over the same time frame. The majority of opioid use in Slovenia can be explained by tramadol in combination with paracetamol, as opposed to the Netherlands where the majority of individuals receive either a prescription for oxycodone or tramadol. The use of strong opioids, especially, oxycodone is very low in Slovenia, whereas in the Netherlands use is high and increasing. One of the reasons for differences in strong opioid use in both countries could be explained by differences in prescribing practice of strong opioids, which is very stringent in Slovenia and much more lenient in the Netherlands. We demonstrated that prescribing strategies of analgesic medication differ substantially between countries in Europe. It is our opinion that the field of guidelines in the treatment of pain warrant further inquiries to be able to achieve consensus in pain treatment and could become a foundation for harmonized guidelines.

## Data Availability Statement

The datasets presented in this article are not readily available because Data obtained in all analyses cannot be shared with third parties as Statistics Netherlands and Health Insurance Institute of Slovenia do not permit this to protect the privacy of patients. Requests to access the datasets should be directed to Ajda Bedene, a.bedene@lumc.nl.

## Disclosure

AD has received research grants and personal fees from MSD, grants from Medasense, grants from Grunenthal, personal fees from Trevena, grants from AMO Pharma, grants from Center for Human Drug Research, outside the submitted work.

## Author Contributions

AB, WL, FR, and ED designed the study. AB and WL had full access to the Statistics Netherlands dataset and analyzed the data. AB drafted the manuscript. All authors provided critical revisions and approved the final submitted version.

## Author Disclaimer

The views expressed in this paper are those of the authors and do not necessarily reflect the views or policies of the NWO. The NWO is not liable for any use that may be made of the information presented.

## Conflict of Interest

The authors declare that the research was conducted in the absence of any commercial or financial relationships that could be construed as a potential conflict of interest.

## Publisher's Note

All claims expressed in this article are solely those of the authors and do not necessarily represent those of their affiliated organizations, or those of the publisher, the editors and the reviewers. Any product that may be evaluated in this article, or claim that may be made by its manufacturer, is not guaranteed or endorsed by the publisher.
