# Supplementary material for: Comparison of Two Different Analgesic Prescription Strategies and Healthcare Systems: Slovenia vs. the Netherlands
Source: Front Pain Res (Lausanne). 2021 Aug 27;2:723797. doi: 10.3389/fpain.2021.723797 (PMC8915570; doi:10.3389/fpain.2021.723797)
Supplement: Supplementary file 1 [file Data_Sheet_1.PDF]

Supplement to: Comparison of two different analgesic prescription strategies and healthcare systems: Slovenia versus the Netherlands

Authors: Ajda Bedene, MPharm[1,2]; Anita Strmljan, MPharm[3]; Eveline L.A. van Dorp, MD PhD[2]; Mitja Udovič, MSc[3]; Willem M. Lijfering, MD PhD[1]; Marieke Niesters, MD PhD[2]; Prof. Frits R. Rosendaal, MD PhD[1]; Prof. Albert Dahan, MD PhD[2]; Jurij Fürst, MD MSc[3]*

Supplementary Table 1. Reimbursed pain medication in the Netherlands and in Slovenia from 2013 to 2019

Supplementary Table 2. (Standardized) prevalence of pain medication use, the Netherlands and Slovenia, 2013-2018(9)

Supplementary Table 1. Reimbursed pain medication in the Netherlands and in Slovenia from 2013 to 2019

ATC	INN	strong/weak opioid	Reimbursed in Slovenia	Reimbursed in the Netherlands
N02AA01	Morphine	strong	yes	yes
N02AA03	Hydromorphone	strong	yes	yes
N02AA04	Nicomorphine	strong	no	yes
N02AA05	Oxycodone	strong	yes	yes
N02AA51	Morphine combinations	strong	no	yes
N02AB02	Pethidine	strong	no	yes
N02AB03	Fentanyl	strong	yes	yes
N02AC01	Dextromoramide	strong	no	yes
N02AC03	Piritramide	strong	no	yes
N02AD01	Pentazocine	strong	no	yes
N02AE01	Buprenorphine	strong	yes	yes
N02AX06	Tapentadol	strong	yes	yes
N02AJ13	Tramadol with paracetamol	weak	yes	yes
N02AX02	Tramadol	weak	yes	yes
M01AA01	Phenylbutazone	NA	no	yes
M01AB01	Indomethacin	NA	yes	yes
M01AB05	Diclofenac	NA	yes	yes
M01AB08	Etodolac	NA	yes	no
M01AB16	Aceclofenac	NA	no	yes
M01AB55	Diclofenac, combinations	NA	yes	yes
M01AC01	Piroxicam	NA	no	yes
M01AC06	Meloxicam	NA	yes	yes
M01AE01	Ibuprofen	NA	yes	yes (not 400 mg)
M01AE02	Naproxen	NA	yes	yes
M01AE03	Ketoprofen	NA	yes	yes
M01AE11	Tiaprofenic acid	NA	no	yes
M01AE17	Dexketoprofen	NA	yes	yes
M01AE52	Naproxen and esomeprazole	NA	no	yes
M01AH01	Celecoxib	NA	yes	yes
M01AH05	Etoricoxib	NA	yes	yes
M01AX01	Nabumeton	NA	no	yes
M01AX17	Nimesulid	NA	yes	no

Abbreviations: ATC, anatomical therapeutic classification; INN, international non-proprietary name

Supplementary Table 2. (Standardized) prevalence of pain medication use, the Netherlands and Slovenia, 2013-2018(9)

**Opioid prescriptions per country**	**Year**	**No.**	**Total No.**	**Prevalence (95% CI)**	**Prevalence (95% CI), standardized**	**RR (95% CI)**	**RR (95% CI), standardized**
Slovenia	2013	155991	2058821	7.58 (7.54-7.61)	7.60 (7.56-7.64)	1 (reference)	1 (reference)
	2014	155961	2061085	7.57 (7.53-7.60)	7.52 (7.48-7.55)	1.00 (0.99-1.01)	0.99 (0.98-1.00)
	2015	155677	2062874	7.55 (7.51-7.58)	7.42 (7.38-7.45)	1.00 (0.99-1.00)	0.98 (0.97-0.98)
	2016	152348	2064188	7.38 (7.34-7.42)	7.17 (7.13-7.21)	0.97 (0.97-0.98)	0.94 (0.94-0.95)
	2017	149592	2065895	7.24 (7.21-7.28)	6.96 (6.92-6.99)	0.96 (0.95-0.96)	0.92 (0.91-0.92)
	2018	140301	2066880	6.79 (6.75-6.82)	6.45 (6.41-6.48)	0.90 (0.89-0.90)	0.85 (0.84-0.85)
	2019	133608	2080908	6.42 (6.39-6.45)	6.04 (6.01-6.07)	0.85 (0.84-0.85)	0.80 (0.79-0.80)
The Netherlands	2013	814211	16779575	4.85 (4.84-4.86)	5.03 (5.02-5.04)	1 (reference)	1 (reference)
	2014	863110	16829289	5.13 (5.12-5.14)	5.26 (5.25-5.28)	1.06 (1.05-1.06)	1.05 (1.04-1.05)
	2015	921763	16900726	5.45 (5.44-5.46)	5.55 (5.53-5.56)	1.12 (1.12-1.13)	1.10 (1.10-1.11)
	2016	975990	16979120	5.75 (5.74-5.76)	5.80 (5.79-5.81)	1.18 (1.18-1.19)	1.15 (1.15-1.16)
	2017	1027019	17081507	6.01 (6.00-6.02)	6.03 (6.01-6.04)	1.24 (1.24-1.24)	1.20 (1.19-1.20)
	2018	1030529	17181084	6.00 (5.99-6.01)	5.97 (5.96-5.99)	1.24 (1.23-1.24)	1.19 (1.18-1.19)
	2019	999699	17282163	5.78 (5.77-5.80)	5.73 (5.72-5.74)	1.19 (1.19-1.20)	1.14 (1.14-1.14)
**NSAIDs prescriptions per country**							
Slovenia	2013	500595	2058821	24.31 (24.26-24.37)	24.16 (24.09-24.23)	1 (reference)	1 (reference)
	2014	501206	2061085	24.32 (24.26-24.38)	24.08 (24.02-24.15)	1.00 (1.00-1.00)	1.00 (0.99-1.00)
	2015	509224	2062874	24.69 (24.63-24.74)	24.40 (24.33-24.46)	1.02 (1.01-1.02)	1.01 (1.01-1.01)
	2016	496742	2064188	24.06 (24.01-24.12)	23.73 (23.67-23.80)	0.99 (0.99-0.99)	0.98 (0.98-0.99)
	2017	502158	2065895	24.31 (24.25-24.37)	23.94 (23.87-24.00)	1.00 (1.00-1.00)	0.99 (0.99-0.99)
	2018	508271	2066880	24.59 (24.53-24.65)	24.19 (24.12-24.26)	1.01 (1.01-1.01)	1.00 (1.00-1.01)
	2019	510234	2080908	24.52 (24.46-24.58)	24.13 (24.06-24.20)	1.01 (1.01-1.01)	1.00 (0.99-1.00)
The Netherlands	2013	2600896	16779575	15.50 (15.48-15.52)	15.78 (15.77-15.80)	1 (reference)	1 (reference)
	2014	2535617	16829289	15.07 (15.05-15.08)	15.29 (15.27-15.31)	0.97 (0.97-0.97)	0.97 (0.97-0.97)
	2015	2469770	16900726	14.61 (14.60-14.63)	14.78 (14.76-14.80)	0.94 (0.94-0.95)	0.94 (0.93-0.94)
	2016	2405557	16979120	14.17 (14.15-14.18)	14.29 (14.27-14.31)	0.91 (0.91-0.92)	0.91 (0.90-0.91)
	2017	2345221	17081507	13.73 (13.71-13.75)	13.82 (13.80-13.84)	0.89 (0.88-0.89)	0.88 (0.87-0.88)
	2018	2294707	17181084	13.36 (13.34-13.37)	13.42 (13.40-13.44)	0.86 (0.86-0.86)	0.85 (0.85-0.85)
	2019	2217218	17282163	12.83 (12.81-12.85)	12.86 (12.84-12.88)	0.83 (0.83-0.83)	0.81 (0.81-0.82)
